# Ambivalent bonds, positive and negative emotions, and expectations in teachers’ perceptions of relationship with their students with ADHD

**DOI:** 10.1080/17482631.2022.2088456

**Published:** 2022-06-16

**Authors:** Arnost Krtek, Klara Malinakova, Ruzena Krtkova Rudnicka, Marketa Pesoutova, Vendula Zovincova, Zdenek Meier, Peter Tavel, Radek Trnka

**Affiliations:** a Sts Cyril and Methodius Faculty of Theology, Olomouc University Social Health Institute, Palacký University, Olomouc, Czech Republic; b The Department of Science and Research, Prague College of Psychosocial Studies, Prague, Czech Republic

**Keywords:** ADHD, teacher, student, relationship quality, factors

## Abstract

A growing body of research has been focusing recently on the life and well-being of students with attention-deficit/hyperactivity disorder (ADHD) and also on the well-being of their teachers. However, there is a need for in-depth, qualitative insights into ADHD issues from the teachers’ perspectives. Therefore, the main aim of this qualitative study was to use thematic analysis to explore how teachers perceive the relationship with students with ADHD and the factors that influence the quality of this relationship. Sixteen teachers working with adolescent ADHD students were interviewed for this purpose. The results indicate that the quality of the teacher-ADHD student relationship is associated with the ADHD students related behaviours, ambivalent emotions of the teacher, the teacher’s beliefs about ADHD and the beliefs about the determinants of the behaviour of the students with ADHD and the teacher’s approaches and methods of work in the classroom. Furthermore, the results suggest that increasing the quality of the teachers’ well-being is associated with knowledge of ADHD determinants, regulation of ambivalent emotions, empathy, teachers’ ability to perceive positive qualities and the potentials of the students with ADHD and their motivation to teach ADHD students.

Attention-deficit/hyperactivity disorder (ADHD) affects approximately 5% of children worldwide (Polanczyk et al., 2007; Polanczyk et al., 2014) and significantly influences the quality of the teacher-ADHD student relationship (Portilla et al., 2014; Rogers et al., 2015) and thus the well-being of the teachers (Spilt et al., 2011) and students with ADHD (Ewe, 2019). The determinants of ADHD are complex, broad, dynamic and ambiguous (see Erlandsson et al., 2016; Perez-Alvarez, 2017). Literature considers mostly neurodevelopmental factors (Dark et al., 2018), genetics (Akutagava-Martins et al., 2016; te Meerman et al., 2019), environmental factors (Martin et al., 2018; Mill & Petronis, 2008; Thapar et al., 2013), culture (Asherson et al., 2012), but also other factors (Sagiv et al., 2013). Based on clinical practice and for diagnostic purposes, the following ADHD subtypes are distinguished: predominantly inattentive (Saad et al., 2018), predominantly hyperactive-impulsive (Sagvolden et al., 2005) and combined type of ADHD (Bluschke et al., 2018).

Theoretically, the quality of the relationship of the teacher with the student with ADHD can be approached through the dimension of closeness and conflict in the interaction between the teacher and the student with ADHD (Mason et al., 2017). The dimension of conflict captures the degree of negative and conflicting relationships and the dimension of closeness involves the degree of acceptance, warmth and care (Zendarski et al., 2020). These dimensions capture both the negative and positive aspects of the quality of the teacher-student with ADHD relationship (Pianta & Stuhlman, 2004).

Another theory that can provide an understanding of the factors influencing the quality of the teacher-ADHD student relationship is labelling theory (Bernburg 2009). Labelling theory assumes that the behaviour and self-identity of individuals can be conditioned by the terms by which they are classified or described. This means that the majority tends to consider minority’s behaviour inconsistent with standard cultural norms to be deviant, which can lead to self-fulfilling prophecy (Jussim, 1986). A self-fulfilling prophecy is a vicious circle in which the pressure of the expectation of others evokes in the individual a reaction that corresponds to the others’ expectations. Thus, if teachers expect disruptive behaviour from ADHD students, the pressure of this expectation may actually trigger that disruptive behaviour of those students (Wiener et al., 2012).

Mirroring this theoretical rationale, various studies showed the importance of closeness-conflict and interaction when exploring the quality of the relationship between teachers and students with ADHD. A book by Pianta (1999) suggested that the teacher-student with ADHD conflict was associated with a disruptive behaviour of students with ADHD, off-task behaviours, difficult classroom management and negative emotional interactions. However, the closeness was related to positive emotional teacher-student with ADHD interactions. A correlational study by Portilla et al. (2014) found that there are more conflicts and less closeness in the relationship of teacher-children with ADHD entering school associated with symptoms of inattention and impulsivity and that the aspects of conflict and closeness in the relationship with teachers were important for children with ADHD. Other correlational studies concluded that teachers hold significantly ambivalent and less favourable attitudes towards students with ADHD (Anderson et al., 2012) and feel less emotionally connected (Rogers et al., 2015). The systematic review by Ewe (2019) suggested that students with ADHD generally feel less close to their teachers compared to other students. Similarly, teachers experience less emotional closeness, less cooperation and more conflicts with ADHD students than with other students.

Correlational studies generally suggested that ADHD students are a factor disrupting the education, learning processes and concentration of teachers and classmates, e.g., by shouting out answers before being called on (Alomar & Strauch, 2014), motoric restlessness (Cortese et al., 2005), sluggish cognitive tempo (Burns et al., 2017), attention decrements (Rapport et al., 2009), conduct problems, hyperactivity (Washbrook et al., 2013) or verbal and physical assaulting of classmates (Velki & Dudas, 2016). A systematic review by Gwernan-Jones et al. (2016) suggested that symptoms of ADHD may be initiated by classroom context that requires students to sit still, be quiet and concentrate. The symptoms of ADHD may then be exacerbated through stigma and damaged self-perceptions of students with ADHD. These experiences further decrease the quality of the teachers-students with ADHD relationships and the students with ADHD-classmates relationship.

A correlational study by Greene et al. (2002) concluded that primary school teachers rated students with ADHD more stressful to teach than their classmates, especially if the students with ADHD exhibit oppositional/aggressive behaviour. A correlational study by Zendarski et al. (2020) suggested that the quality of the student-teacher relationship was associated with medication use, premature sexual activity among ADHD students (child sex), ADHD subtype, cognitive/academic functioning and behaviour of students, the teachers’ years of experience, self-efficacy, parental education and socio-economic status. Zendarski et al. (2020) also found out that the ADHD students experience a poorer quality of a student-teacher relationship compared to students without ADHD and that the poorer quality of this relationship was associated primarily with the behavioural problems of ADHD students and the teachers-students ADHD conflicts when teachers try to solve early child sex (sexual conduct problems and sexual behaviour that is inappropriate for the age of the children).

A correlational study by Masse et al. (2015) emphasized the students’ problematic behaviour to be a major source of teachers’ stress in general. A qualitative study by Indri Hapsari et al. (2020) found out that the teachers’ relationship with ADHD students is negatively influenced by the teachers’ lack of knowledge. The teachers were not aware that students with ADHD have several problems, including a problem with their self-perception, a problem in social relation, academic problems, problems with negative behaviour, and a negative label from their surroundings. In contrast, a qualitative study by Wienen et al. (2019) suggested that the classification of ADHD in students changes the perception of determinants of the disruptive behaviour of these students. The determinants of ADHD are beginning to be perceived in the disorder instead of the failure of ADHD students, parents and teachers. This shift in perception of ADHD removes mutual blaming and feelings of guilt from the teacher-ADHD student relationship, which improves the quality of that relationship and mutual cooperation.

Current literature also draws attention to the risks for students with ADHD and teachers that result from a disrupted teacher-student relationship. A systematic review by Ewe (2019) emphasized that the teachers’ rejection of ADHD students can contribute to school failure, peer exclusion and rejection, which can lead to a low self-esteem and a sense of loneliness for students with ADHD. A qualitative study by Ahlström and Wentz (2014) pointed out that these negative consequences and other difficulties in everyday life may be potential factors reducing the well-being of those young persons with ADHD. A systematic review by Brunsting et al. (2014), and a qualitative study by Corbin et al. (2019) suggested that the poor quality of the teacher-student relationship and the high workload that working with students with special educational needs requires may also lead to the teachers’ emotional exhaustion and burnout syndrome.

A correlational study by Greene et al. (2002) pointed out that teaching students with ADHD is a big challenge, as these students require a significantly higher percentage of the attention than students without ADHD. Nevertheless, as a correlational studies by Baker (2006), Roorda et al. (2011) and a systematic review study by Bergin and Bergin (2009) suggested, a teacher should provide supportive attitudes and behaviour that can increase engagement with school, well-being, optimal adjustment and secure attachment of students with ADHD. Bowlby’s book review by Rutter (1989) suggested that promoting a secure attachment means that the important others are sufficiently available and responsible, which allows children to build a sense of security that is a safe basis, facilitating a healthy psychosocial development and exploration of the world. A study by Verschueren and Koomen (2012), which brought together theoretical literature and empirical studies, suggested that the teachers can contribute to the formation of the students’ secure attachment through sensitivity to the students’ needs. Teachers’ sensitivity to the students’ needs may also significantly improve the quality of the teacher-student relationship. This is especially important for students with ADHD who tend to have rollercoaster relationships and form a more insecure attachment associated with family separation anxiety and school separation anxiety than their non-ADHD peers, as suggested by a qualitative study by Moore et al. (2017). A review study by Bergin and Bergin (2009) suggested that a good teacher-student with ADHD relationship and a secure attachment of students with ADHD are factors associated with greater emotional regulation, social competence, lower crime rates, and a willingness to accept challenges, which is essential for school achievement. A correlation studies by Corbin et al. (2019) and Spilt et al. (2011) found that the quality of the relationship between the teacher and the student is also associated with higher levels of the teachers’ personal accomplishment over the academic year and the professional and personal self-esteem and thus with the quality of the teachers’ well-being. Similarly, a correlational study by Milatz et al. (2015) suggested that the quality of the teachers’ well-being is enhanced by their secure attachment and the quality relationship with ADHD students.

## The present study

An increasing number of quantitative studies in the field of education addressed the teacher-ADHD student relationship and the factors that influence the quality of that relationship. A limitation of those studies may be that the richness of their findings is given by the quantitative nature of the research design. Another limitation can be that the findings of quantitative studies present only the association between factors and the degree of conflict and closeness in the teacher-student with ADHD relationship. Thus, there is still a lack of more detailed insight into factors influencing the quality of the teacher-ADHD student relationship from the lived experience of teachers. Moreover, there is also a need to closely explore how teachers‘ personal beliefs about ADHD students are formed and how those beliefs affect teachers‘ relationship to ADHD students, as suggested, e.g., a qualitative study by Russell et al. (2019).

The importance of a closer understanding of the factors influencing a teacher’s beliefs about students was demonstrated in some recent qualitative studies. Russell et al. (2016) found hierarchical interconnectedness of factors influencing teachers’ beliefs in a teacher-adolescent trust, and that those teachers’ beliefs are not static and must be reinforced over time in positive social interactions between teachers and their students. Jiang et al. (2019) pointed out that teachers’ beliefs about teacher-student power relations can be related with teachers’ appraisals of students’ misbehaviours and that teachers should discuss the problem with students rather than have emotional outbursts or suppress their emotion when they feel a need to direct-stage anger. Sato et al. (2007) explored the beliefs of Japanese physical education teachers about teaching students with disabilities. The main themes identified in those teachers’ beliefs were: communication, collaboration and support, satisfactions, ambivalences and concerns, and professional preparation inadequacies. Russell et al. (2019) found that educational practitioners believe that the disruptive school behaviour of ADHD childrenare determined by the home lives of those children, which are characterized by inconsistency, social isolation and psychosocial adversities such as drug abuse or domestic violence.

From the findings of the aforementioned studies can be identified that a qualitative analysis of teachers’ lived experiences contributes to a deeper and more detailed understanding of the interconnectedness, multi-layered dynamism and ambivalence of factors that influence teachers’ beliefs about students. All these past insights open new avenues for further in-depth investigation of factors influencing teachers’ beliefs about ADHD students and the quality of the teacher-ADHD student relationship.

Therefore, the aim of the present study was to thematically analyse interviews with Czech school teachers to gain more detailed understanding of lived experiences of teachers of ADHD students with teachers’s beliefs and other factors that influence the quality of teacher-ADHD student relationship. The present study also focuses to examine the general assumption that teachers consider the teacher-ADHD student relationship to be more conflictual and less close.

According to previous evidence, we also expected that the quality of the teacher-ADHD student relationship would be influenced primarily by the behaviour and school performance of ADHD students, teachers’ personal beliefs about ADHD, and training and familiarity with ADHD. We also expected that participants would report on practical implications and certain aspects of ADHD students’ personalities that contribute to increasing the quality of the teacher-ADHD student relationship.

## Method

### Measures and procedures

Our study was inspired by the DIPEx methodology (Database of Personal Experiences of Health and Illness), which was developed by researchers at the University of Oxford (Ziebland & McPherson, 2006). The aim of DIPEx methodology is to gain an insight into the lived experiences of people with various illnesses and with professional health care.

DIPEx methodology includes the following main phases: (a) study of scientific literature, (b) compilation of interview schedule and advisory panel, (c) pilot study, (d) clarifying of interview schedule, study plan and participant recruitment strategy, (e) participant recruitment, (f) data collection, (g) data coding, data analysis (thematic analysis), (h) production of the final report. The final report of DIPEx provides a thematically structured lived experience of participants, e.g., how the disease affects family life, work, education and leisure, how to manage the disease or how professional care can be improved. The acquired knowledge is used in health policy and can influence changes in health or social care.

The present study followed the qualitative approach aiming to assess the experience of participants, as described by O’Connor and McNicholas (2020). These authors explored the first-hand lived experience of participants in the context of child and adolescent psychiatry.

Prior to recruiting participants, a semi-structured interview schedule was developed. The interview schedule was designed as an aid to researchers to remind them of the important themes that the participants may not have mentioned on their own. Interview schedule items were constructed based on recent studies concerning ADHD (Brock et al., 2009; Feranska, 2018; Greene et al., 2002; Rushton et al., 2020), especially in the context of school (for other literature see Supplement 1.). The interview schedule items were formulated as open-ended questions, but there was a space for flexibility to create variants of pre-prepared questions or completely new questions as the participants introduced new ideas. The main items of the interview schedule were (a) free storytelling about ADHD, (b) teachers’ attitudes, (c) manifestations of student behaviour, (d) teacher-student relationship, (e) teachers’ education about ADHD, (f) coping strategies, (g) class collective, (h) communication with parents, (i) teaching assistants, (j) school and colleagues, (k) medication, (l) spiritual aspects, and (m) messages to others.

The interview schedule design was inspired by Blank (2013), Majid et al. (2017), and Wood (1974). The questions of the interview’s schedule were piloted and validated through three phases before their use in the major study. First, the researchers sought to construct questions in a way that would lead participants to the report in accordance with the main study objective. In this sense, the important question was “How do you generally feel in a relationship with ADHD students?”, that was included in the interview schedule item “teacher-ADHD student relationship”. Second, the members of the advisory panel revised the relevancy and language quality of the interview schedule questions. Third, the revised questions were tested in the pilot study, in which 4 participants participated (the pilot study’s participants did not participate in the main study). These participants were selected in accordance with the criteria for selecting participants for the major study. Researchers also asked the participant of the pilot study which questions in the interview schedule required further clarification. The aim of the pilot study was also to allow researchers to test and improve their interviewing skills with teachers of ADHD students.

### Participants

The study sample consisted of 16 school teachers of students with ADHD aged 12–14 years (median age 13 years) from all regions of the Czech Republic. For a detailed description of the study sample, see, Table 1.

**Table 1.** Demographic status of teachers working with students with ADHD

Age group	Sex	Pseudonym	Marital status	Education	Years of teachers’ experience	Age of students with ADHD	Subjects taught by teachers
30–39	Woman Woman Woman Woman Man Man	Marta Alena Hana Petra Josef Ales	Married Married Divorced Single Married Divorced	Higher education Higher education Higher education Higher education Higher education Secondary education	5 6 5 5 7 5	12–14 12–14 12–14 12–14 12–14 12–14	Biology Czech language, English Mathematics, Chemistry Czech language, History Czech language, Social Sciences Social Sciences
40–49	Woman Woman Woman Woman Man Man Man	Marika Evelina Katka Ivana Martin Vendelin Matej	Married Married Married Divorced Married Married Married	Higher education Higher education Higher education Higher education Higher education Higher education Higher education	12 6 15 12 8 6 17	12–14 12–14 12–14 12-14 12-14 12-14 12-14	Czech language, Social Sciences Music, English Biology Physics, Chemistry Social Sciences, History Mathematics, Chemistry
50–59	Woman Man Man	Marie Milos Antonin	Married Married Married	Higher education Higher education Higher education	22 15 18	12-14 12-14 12-14	Social Sciences, History Czech language, History Geography

The inclusion criteria for participation in the study were: teachers working with students with ADHD aged 12–14 years, at least 2 years of teaching experience in teaching students with ADHD, teachers were informed about the diagnosis of ADHD by the parents of the students with ADHD, ADHD in students diagnosed by clinical psychologists in accordance with DSM-5 (Lee et al., 2020). Thus, the teachers were not selected on the basis of whether they knew much or little about ADHD. According to the Czech educational system standards, all the teachers underwent only common seminars presenting basic information for working with ADHD students.

The participants were recruited both by direct (i.e., through advertisements in relevant periodicals) and indirect (i.e., the snowball sampling) recruitment strategy. Direct recruitment was carried out through advertisements in periodicals of magazines. The indirect recruitment method was based on the snowball method (Noy, 2008). It was carried out only as a marginal and complementary strategy in which the members of the advisory panel were asked to contact potential participants they knew from their professional practice. The advisory panel was composed of professionals who have experience working with children with ADHD, i.e., doctors, psychiatrists, teachers, special pedagogues, teaching assistants, psychologists, but also students with ADHD and their parents. Other members of the advisory panel were the researchers leading the project, and the project supervisor.

The snowball method was used in our research with full knowledge of its advantages, disadvantages (Audemard, 2020; Lee & Spratling, 2019) and potential biases. So-called “community bias” was considered particularly problematic. This bias means that the researcher or participant may tend to recruit new participants from a particular subgroup and there is no branching out of that narrow community (Sadler et al., 2010). In the present study, this bias was reduced by the primary use of direct recruitment and by the fact that all members of the advisory panel helped with searching for participants.

The snowball method had two phases. In the first phase, a sample of suitable participants was identified and contacted, and in the second phase, the participants identified in the first phase helped to recruit other suitable participants until the number of participants in the sample was satisfactory.

### Data collection procedures

The interviews in our study were conducted by three experienced researchers, who were part of the project team members. They had many years of experience in qualitative research and psychotherapy and underwent professional training in conducting interviews within the DIPEx methodology. The place and time of the interviews were adapted to the possibilities of the participants. The interviews were recorded in the works or homes of the participants in rooms without the presence of other people and distractions. For a more detailed description of the date and place of recording of interviews with participants, see, Table 2.

**Table 2.** Date and place of recording of interviews with participants

Pseudonym of participants	Date	Place
Marta	10/20/2019	A room at participant’s home without the presence of other people and distractions
Alena	7/23/2018	A room at participant’s work(teachers’ room) without the presence of other people and distractions
Hana	10/4/2018	A room at participant’s home without the presence of other people and distractions
Petra	9/14/2018	A room at participant’s home without the presence of other people and distractions
Josef	2/6/2020	A room at participant’s work(classroom) without the presence of other people and distractions
Ales	1/23/2019	Tennis club room without the presence of other people and distractions
Marika	4/23/2020	A room at participant’s home without the presence of other people and distractions
Evelina	3/19/2019	A room at participant’s work(teachers’ room) without the presence of other people and distractions
Katka	4/23/2019	A room at participant’s home without the presence of other people and distractions
Ivana	12/4/2019	A room at participant’s home without the presence of other people and distractions
Martin	10/4/2019	A room at participant’s work(classroom) without the presence of other people and distractions
Vendelin	6/11/2018	A room at participant’s work(laboratory) without the presence of other people and distractions
Matej	11/18/2019	A room at participant’s home without the presence of other people and distractions
Marie	6/12/2019	A room at participant’s home without the presence of other people and distractions
Milos	12/8/2018	A room at participant’s home without the presence of other people and distractions
Antonin	2/15/2019	A room at participant’s home without the presence of other people and distractions

The average length of the interviews was 56 minutes. The interviews were audio and digitally recorded. During the transcription process, the participants were assigned a pseudonym and care was taken to remove all personally identifiable information that could identify actual people, children, or places when using data extracts. The transcribed interviews were checked and anonymized in accordance with the DIPEX methodology.

### Data analysis

For the data analysis, the method of thematic analysis was used (Jordan, 2018), because it was suitable for the organization and descriptions of the identified themes in the data. This way of processing data is suitable for understanding the teachers’ experiences regarding the quality of the relationship between the teacher and the student with ADHD. The focus on the quality of the teacher-ADHD student relationship arose from the finding that the participants devoted the most time to this theme in the interviews and gave the greatest importance to it.

Another benefit of the thematic analysis is that it allows the identification of the similarities and differences in different perspectives of the participants, the new insights into the issue and the summary of the key themes and their structuring into a final report (Nowell et al., 2017). The thematic analysis has several phases, which involve familiarizing researchers with data, generating initial codes, and searching, reviewing, and defining themes. Familiarizing researchers with data usually takes place through transcribing audio recording, reading through the text and taking initial notes, and generally looking through the data. The coding phase involves highlighting parts of the text and creating labels or “codes” that concisely express the content of the parts of the text. The phase of searching, reviewing and defining themes involves bringing the codes into clusters and finding thematic connections between the clusters and the codes (Braun & Clarke, 2014).

Prior to our thematic analysis, the researchers repeatedly and carefully read the transcripts of the interviews. Three researchers projected each transcript on a projection screen and read it together in the next step. The text of the transcripts was marked by thematic codes and sub-codes (names of more generally emerging themes and subthemes), such as “working with a student with ADHD”, “students with ADHD”, “symptoms of ADHD”, etc. Some passages of the texts were marked with multiple codes because they contained multiple meanings. The resulting list of thematic codes and sub-codes was then assessed using the NVIVO 12 program. Researchers made a node of the same name for each thematic code and inserted the appropriate sub-nodes for each. For example, under the node “student with ADHD” there were sub-nodes “problems of a student with ADHD”, “self-perception of student with ADHD”, “relationships of students with ADHD”. In the second phase, the transcript files of the interviews were uploaded by researchers to the program NVIVO 12. Consequently, researchers sorted and inserted the text of the transcripts under the relevant nodes and sub-nodes. At this phase, the researchers continuously discovered some other sub-nodes (sub-codes) that enriched the already existing tree of sub-nodes. For a detailed description of the system of thematic codes (nodes) and sub-thematic codes (sub-nodes), see, Table 3.

**Table 3.** System of thematic codes (nodes) and sub-thematic codes (sub-nodes)

Thematic codes (nodes)	Sub-thematic codes (sub-nodes)
Students with ADHD	Problems of students with ADHD Self-perception of students with ADHD Relationships of students with ADHD
Teacher about his work	Teachers’ needs Perception of ADHD Advice and recommendations for working with ADHD students Relationship to students with ADHD Coping with students with ADHD
Working with a student with ADHD	Communication with students with ADHD Motivation of working with students with ADHD Challenges of working with with students with ADHD
Symptoms of ADHD	Aggression Emotion Hyperactivity Impulsivity Positive aspects of ADHD Attention Symptom changes
Parents	Communication with parents of students with ADHD

Subsequently, the researchers read the texts assigned under the nodes and sub-nodes. They wrote the participants’ statements out on a clean paper and marked them with identification labels to visualize which participants were involved. Next, the statements were cut and, based on their relation, placed side by side. Thus, the researchers created several clusters and labelled them by abstracting a more general meaning of the statements. Furthermore, the researchers looked for thematic connections between the statements within one cluster and thematic connections between clusters and between the statements of different clusters. For a detailed description of the frequency of themes, see, Table 4.

**Table 4.** Frequency of themes

Theme	Number of participants reporting the theme
Teachers’ strong ambivalent bond with students with ADHD	15
Teachers’ negative emotions in relation to students with ADHD	15
Negative emotions of teachers due to repeated negative experiences with students with ADHD	8
Teachers were relieved not to have to teach students with ADHD	4
Teachers’ experience that students with ADHD disrupted teaching	12
The feeling of helplessness of teachers as a result of the behaviour of students with ADHD in the classroom	3
Positive emotions of teachers towards students with ADHD	15
Positive emotions of the teacher in response to some specific abilities of students with ADHD	7
The teachers’ beliefs that the students with ADHD are abusing the diagnosis of ADHD, which led to the teachers’ lower tolerance and a sense of closeness to students with ADHD	2
The teachers’ beliefs that disruptive behaviour of the students with ADHD is unintentional, which led teachers to increase tolerance and a sense of closeness to students with ADHD	6
The teachers’ beliefs that some disruptive behaviour of the students with ADHD is unintentional and other disruptive behaviour is intentional, which leads teachers to increase tolerance for disruptive behaviour considered unintentional and not to accept disruptive behaviour considered intentional.	8
How can teachers positively influence the quality of a teacher-student with ADHD relationship	16
Mental preparedness for disruptive behaviour of students with ADHD. Pre-prepared prompt but calm interventions.	16
The teachers offered students with ADHD to work together to improve their relationship.	7
The teachers explained to the student with ADHD the symptoms of ADHD and that it is possible to learn to respond to the symptoms with more adaptive behaviour, which they can try to find and train together.	3
The teachers tried to be a role model for students with ADHD in the performance of duties	2
The teachers built a mutual trust with the students with ADHD, which enabled open communication about the symptoms of ADHD and the current needs of students with ADHD	5
The teachers noticed and appreciated the strengths and specific abilities of students with ADHD. The teachers used the strengths and special abilities of students with ADHD to streamline the learning process in the classroom. In this way, there was an increase in self-esteem of students with ADHD, increased closeness in the teachers-students with ADHD relationship and a better social status of students with ADHD in the classroom.	7 4

The structure and content of the results were based on these themes. The starting point of the result´s structure was chosen on the basis of the theme “the teacher’s strong ambivalent bond with students with ADHD”, because this theme emerged explicitly and implicitly from all clusters. This theme also became the label of the first subsection of the results and included the themes “negative emotions in relation to students with ADHD” and “positive emotions in relation to students with ADHD”. The following subsections of the results are “ADHD as an excuse for inappropriate behaviour” and “the behaviours of children with ADHD perceived intentionally or unintentionally,” which present the content of the theme “experiences and beliefs influencing the quality of the teacher-student with ADHD relationship”. The final subsection of the results is the subsection “Improving the quality of the teacher-student with ADHD relationship”, which provides a description of the themes of the same name.

The researchers compiled the structure of the results in such a way that the reader first gains insight into the factors that influence the ambivalent quality of the teacher-student with ADHD relationship and then into the factors that allow the improvement of the quality of the teacher-student with ADHD relationship.

### Ethics

The research was guided by ethical rules and standards based on the DIPEX methodology of Oxford University. The ethical approach of the research was further ensured by the approval of the Ethics Committee of the Cyril and Methodius Faculty of Theology in Olomouc, number (2018/01), by training of all members of the research team on ethical issues, consulting on ethical issues with a supervisor, ethical experts at the university and the advisory panel. The advisory panel was also set up to help researchers refine participant recruitment strategies, interview schedules and the research process.

First, the participants were informed of their rights in the research and they signed informed consent. Informed consent procedures involved written information sheets that were given to participants, verbal information, and opportunities to ask questions prior to participation in the study. Participants were also informed that they can withdraw from the study at any stage and that they will have an opportunity to authorize an anonymized version of texts. Next, the participants were given the researchers’ contact details and received reimbursement for their time.

Regarding the handling of the information, the audio recordings and the transcripts were stored securely in accordance with relevant legal requirements and with current Czech and European legislation on personal data protection (Freitas et al., 2017; Verschuuren et al., 2008).

Conducting some interviews was mentally exhausting because researchers had to work in various home or work environments of the participants and conducted interviews in which participants reported difficult themes. Therefore, psychotherapeutic support was also available to the researchers.

## Results

The findings suggest a strong ambivalent teachers’ bond with students with ADHD, filled with negative and positive emotions influencing the quality of the teacher-student with ADHD relationship and so influencing the well-being of the teachers and the students with ADHD. These emotions were associated with the teachers’ experiences with ADHD students, beliefs about the causes of the ADHD students’ behaviour, and other attitudes and practical approaches of teachers.

### Strong ambivalent bond with students with ADHD

Most participants reported ambivalent emotions in relation to students with ADHD, but also about a strong emotional bond. Participant Ales described these strong and ambivalent emotions in his relationship to a student with ADHD as follows:

*“He is an amazing boy, I sometimes love him very much and I know he loves me too, but when it comes to him, he is unbearable and endangers other children. Our relationship is neither positive nor negative, but it is definitely stronger.”*

### Negative emotions in a relationship with a student with ADHD

In some cases, problems with students with ADHD accumulated so much that negative emotions prevailed and the participants began to have negative expectations about the students with ADHD. The participants may have started to experience the relationship with the students with ADHD negatively because their belief that the behaviour of the student with ADHD must gradually improve was not fulfilled. One of the participants who reported in this sense was Martin:

*“If there are a lot of problems with a child with ADHD and they are all getting worse, then the feelings are only negative. If it doesn’t improve, the relationship gets worse and worse. And I have to say that after a long time I had mostly negative feelings towards the child and sometimes I just hated him*.”

Another participant Marie expressed relief from anxiety when one student with ADHD withdrew from her optional subject:

“*Although I quite like the student with ADHD, I was really relieved from the anxiety when the student withdrew from my music education.”*

Several participants described various situations where the disruptive behaviour of the students with ADHD negatively influenced the teacher’s lecture and the concentration of the other students. In this sense, Ales reported the following:

*“I teach in two classes where there are children with ADHD. There is one boy in each class with quite a strong attention deficit disorder. Both are very disturbing factors, they often interrupt me and try to attract my attention and the attention of the other students whenever they get a chance. It happens that some students, who are normally calm, get seduced by this and start to disturb as well.”*

The participants perceived the students with ADHD to be unable to concentrate during the whole lesson, their motor restlessness to increase gradually, and sometimes to be unpredictable or even dangerous. In such cases, some participants, such as Katka, experienced helplessness and insecurity from losing control over the situation in the classroom:

*“They can do almost anything at any time. It’s totally unpredictable. When that happens, the class is usually surprised because no one knows what’s going on. Sometimes I really have no idea what to do.”*

The participants admitted that when these situations frequently recurred, they felt increasing anxiety during the progress of the class. They felt trapped and without any possibility to handle the chaotic and confusing situation induced by the students with ADHD.

### Positive emotions in a relationship with a student with ADHD

Other participants reported a predominance of positive emotions in relation to students with ADHD. They enjoyed the liveliness, creativity and spontaneity of these students. These experiences had also Josef, who reported:

*“I like them, they are much better than passive children. I like their liveliness, spontaneity and creativity.”*

A few participants considered the relationship with the students with ADHD to be mostly good because they enjoy the sense of humour of the students with ADHD. Antonin reported how he enjoyed the jokes of one ADHD student:

“*My relationship with the students with ADHD is great, perfect! It’s a lot of fun with them. They have the skill to make fun and jokes*.”

Another participant Hana emphasized, that her relationship with a student with ADHD was characterized by closeness because she admired the student’s painting talent.

### ADHD as an excuse for inappropriate behaviour

Some participants reported about their experiences and beliefs that some parents and their children (students) with ADHD overused the diagnosis of ADHD. In a way, such labelling allows students diagnosed with ADHD not to accept guilt for the negative consequences of their disruptive behaviour because it is believed that ADHD causes the disruptive behaviour. Participant Milos complained that some parents and their children with ADHD constantly made excuses that their child cannot be blamed for inappropriate behaviour because it is caused by ADHD. Parents and ADHD students were also hiding in the role of victims of bad behaviour by classmates, bad teachers’ methods that cause their child to behave inappropriately so that their child would not have to be held responsible for his behaviour and his parents for poor parenting. Milos described his experience and beliefs about the abuse of ADHD by some ADHD students and parents as follows:

*“They’re just hiding behind it. Do they say what you want from us? We were diagnosed with ADHD. They play the role of the victim and say that the teacher uses bad methods and other children treat a child with ADHD ugly, which is why a child with ADHD reacts so aggressively. These parents try to hide their parental failure behind ADHD.”*

Several participants, such as Ivana, also reported abusing the diagnosis of ADHD by a student:

*“He knows everything about his diagnosis and acts as if his ADHD entitles him to do whatever he wants and not learn anything he doesn’t want. He thus successfully avoids school duties and learning.”*

The participants with this type of experience and beliefs about the students with ADHD and their parents more often reported a feeling of less closeness and tolerance in their relationships with those students because they did not believe that disruptive ADHD students’ school behaviour was conditioned by ADHD. These participants considered the personality of ADHD students to be spoilt by poor parenting. This means that those students deliberately avoid responsibility and put intentional resistance to educational efforts because they are used to doing only entertaining activities that bring them immediate satisfaction.

### The behaviours of children with ADHD perceived intentionally or unintentionally

Some participants reported experiences and beliefs that ADHD students understood their instructions and knew that their behaviour was inappropriate, but they are not able to control the behaviour and to behave according to the rules due to a higher level of impulsivity. In this sense, Ivana reported:

*“I feel that they know me well and know what bothers me and what they should not do. I’m sure they know that if they do something wrong, trouble will come. To be honest, I’m not convinced they can’t help themselves. They have no emergency brake.”*

The participants with these types of experiences and beliefs reported more closeness in the teacher-student with ADHD relationship and their efforts to tolerate and understand the students with ADHD and to empathize with them, because they perceived the disruptive behaviour as unintentional and as a consequence of the disorder, not as part of the students’ personality.

A few participants expressed the belief that certain behaviour in children with ADHD was undoubtedly the symptoms of ADHD, and different behaviour was completely intentional and the diagnosis of ADHD was misused as an excuse. Evelina commented on this matter as follows:

“*I know that his behaviour is a symptom of ADHD, usually only after the outburst of a stronger rage. This does not happen with my own children who do not have ADHD. But then I also know of an ADHD boy who abuses the diagnosis of ADHD and claims that he can’t do any work because of it.”*

The participants with this type of belief often tried to distinguish between the boundaries of the behaviour caused by ADHD, and the behaviour caused by poor parenting. According to the participants’ personal opinion on, the distinction between behaviour caused by ADHD and by poor parenting may help to reduce conflicts in the relationship between the participants and their ADHD students. The conflict was reduced because the teachers stopped blaming the ADHD students for the ADHD-conditioned behaviour.

### How to positively influence the quality of the teacher-student with ADHD relationship

Most of the participants reported that they experience ambivalent emotions towards students with ADHD. Negative emotions were often evoked by the fact that working with students with ADHD was demanding and consuming a considerable amount of energy. Petra expressed this as follows:

*“Working with him (ADHD student) was frustrating. I gave him a thousand percent of my attention and that’s completely exhausting. It happened to me that I blew up in a rage a few times, because you are doing your best and there are still problems and incidents with him, and that is annoying.”*

Other participants reported that the desire of the students with ADHD for a teacher’s attention is bottomless. Some teachers pointed out that they often feel overwhelmed by this, because they also have to work with thirty other students in the classroom.

The participants emphasized that despite the strong negative emotions that students with ADHD sometimes evoke, it is necessary for the teacher to try to remain calm and think a few steps ahead. Such a teacher’s approach has the advantage of not destroying the closeness of the teacher-student with ADHD relationship through excessive negative emotions of the teacher and acute conflict. One of these participants reporting in this sense was Marta:

*“Children with ADHD often surprise me and I am upset, but I try to think ahead. When I go to this lesson to meet this child, I have to mentally prepare for it and I have to tell myself, the problem may come, stay calm, you have to solve it calmly. You can’t yell at him, it won’t help him anyway, it won’t help anyone.”*

With this attitude, the teachers set their consciousness to a “standby mode” in which they expect that the disruptive behaviour of a student with ADHD to occur, and that such behaviour needs to be responded to quickly but calmly. This way of mental preparedness allows teachers to alleviate their own shock from unexpected events, to better regulate strong emotions and also prevent the stress of a student with ADHD from a possible over-emotional reaction of the teacher. Other participants reported that their relationship with a student with ADHD improved when they agreed with a student with ADHD on what to do in order for the collaboration to work better. Josef expressed this as follows:

“*It was really challenging and exhausting with him. It helped that I told him that we spent more time at school together during the week than his parents spent at home with him, and that together we had to figure out how to do it to feel better and work better together.”*

Such teachers’ approach is characterized by an offer of closeness and mutual responsibility in the teacher-student with ADHD relationship, teacher acceptance of a student with ADHD and an offer of more equal cooperation. Several participants were able to reduce conflicts in the teacher-student with ADHD relationship by explaining to an ADHD student that it is possible to respond to ADHD symptoms with more adaptable behaviour and also by trying to be a positive role model. Josef reported in this sense as follows:

“*The number of conflicts reduced when I explained to him that ADHD did not cause him to be unable to perform his duties, but that ADHD was only about being inattentive and in inner tension. It is therefore necessary to find a way to work with inattention and tension. I also tried to be a role model for him and honestly perform my duties*.”

Furthermore, the quality of the teacher-student with ADHD relationship improved when the participants allowed the student to communicate openly about current needs. For example, one participant entered into an agreement with a student with ADHD based on mutual trust that a student with ADHD could truthfully tell that he was already overwhelmed by learning. In this case, the participants allowed the student with ADHD to walk or run down the hall. When the student relaxed, he returned to class. Most participants also reported that the quality of the teacher-student with ADHD relationship tends to gradually improve after several years of mutual cooperation because the teacher and the student get to know each other’s strengths and weaknesses and thus can respect each other more. Alena also described this as follows:

“*It took us about two years to understand what we can expect from each other, what works, what doesn’t work, and where we need to refine our cooperation*.”

This mutual attitude presupposes the will to try to re-establish closeness despite recurring conflicts.

Some participants sought to improve the relationship with the students with ADHD by deliberately drawing attention to the positive skills of the students with ADHD. When teachers were able to take advantage of the positive qualities of a student with ADHD in behaviour of teaching the whole class, the quality of the teacher-student with ADHD relationship improved. This teacher’s approach also improved the self-esteem of the students with ADHD and class social status of the student with ADHD. Marie reported in this sense as follows:

“*I tried to focus not only on the bad, but above all on the good. Praise him for it. Point it out. Show him that you also notice what he can do. One of those ADHD boys could draw beautifully. So I used it. When we were discussing a topic in a subject, I asked him to draw something on that topic. And he drew it very beautifully. And then he knew that I was interested in him and also that I would exhibit the pictures. And thanks to that, he knew that he was not just the boy who was naughty, he already knew that he could do something, and his classmates began to take him seriously and appreciate him.”*

Some participants reported that their motivation to work on a good relationship with a student with ADHD is based on the joy of being able to make some progress and success with these students. Furthermore, the motivation is based on the desire to manage the challenge of working with ADHD students, to better understand the hidden causes of ADHD and to be a good professional. Marika put it this way:

“*I feel satisfaction from it. Although the result is never completely perfect, I enjoy the work. And every little success does me good. It makes me happy to be able to work with children with ADHD.”*

Two participants described their experiences with children with ADHD very confidently and conceitedly. They argued that they mastered the teaching of students with ADHD flawlessly and reported that they excluded all emotionality from the relationship with the students with ADHD. They also did not mention any conflicts with students with ADHD. These participants gave the researcher the impression that they were trying to protect their low professional self-esteem. Matej reported this in this way:

*“I graduated from three universities focused on special pedagogy and I work perfectly with children with ADHD, I do not have any problems. And how do I experience a relationship with students with ADHD? I don’t experience it. It doesn’t make sense to deal with emotions. All you have to do is to do things right.”*

## Discussion

The aim of this study was to assess the teachers’ perceptions of quality of their relationship with ADHD students and to gain insight into the factors that influence the quality of this relationship. We found that teachers often perceive ambivalent emotions to their ADHD students. Furthermore, our findings suggest that the quality of the teacher-ADHD student relationship is mainly influenced by the students´ behaviour, teachers’ beliefs and knowledge of ADHD, special abilities and talents of ADHD students, teachers’ respect to special educational needs and teachers’ motivation and resilience.

### Positive, negative and ambivalent teachers´ emotions

Although most participants in our study reported positive emotions in relation to students with ADHD, the strength of the negative emotions was tangible, which is in line with the findings of other authors (Ewe, 2019; Zendarski et al., 2020). Ewe (2019), Zendarski et al. (2020) suggested the poorer quality of the relationship between a teacher and a student with ADHD than between a teacher and a student without ADHD. There are several possible explanations for these findings. First, the participants’ negative emotions, such as anxiety, helplessness, anger, fatigue, and exhaustion, were found to be their responses to unpredictability, hyperactivity, inattention, motor restlessness, and resistance to authority in students with ADHD, which is in line with other studies (Greene et al., 2002; Masse et al., 2015). These experiences might result in higher levels of stress in teachers, as described e.g., by Greene et al. (2002), who reported that teachers of ADHD students experienced a three times higher level of stress compared to other teachers. In our study, some participants explained this situation by the long-term accumulation of stressful situations with ADHD students, suggesting that at some point these experiences may form an internal generalized negative attitude towards these students. Negative emotions may gradually overshadow the positive experience, and the teacher creates a strong association between the negative emotions and the idea of working with students with ADHD. Second, some factors contributing to the negative emotions of the teachers towards ADHD students may in fact lie on the side of the teachers. These factors may involve e.g., teacher’s burnout syndrome (Corbin et al., 2019; Hoglund et al., 2015) or the insecure attachment style of the teacher (Morris-Rothschild & Brassard, 2006; Sher-Censor et al., 2019). Third, other factors may be of a more practical nature, e.g., the unanswered need for regular teachers’ supervision (Hoque et al., 2020; Mogg, 2020), the lack of cooperation between school management and parents (Feranska, 2018), the lack of extra educational support for ADHD students (De Boer & Kuijper, 2021), too many students in the classroom or teachers’ lack of understanding or misinformation of the symptoms of ADHD (te Meerman et al., 2017).

Nevertheless, in our study, the participants described their relationship with ADHD students also in positive terms, such as joy, compassion, and love. These testimonies described how the teachers enjoyed the liveliness, creativity, authenticity, sense of humour and artistic talent of the students with ADHD. This is consistent with Sedgwick et al. (2019), who consider divergent-thinking, non-conformity, hyper-focus, adventurousness, self-acceptance and sublimation of excessive energy, as positive aspects of students with ADHD. These authors also pointed out that while the excessive amount of uncoordinated energy hidden in hyperactivity can be disruptive for an ADHD student and classmates during the learning process in the classroom, this energy can turn into a liveliness leading to productive ends when sublimed into the activity that ADHD students love. The close relationship and the teachers´ positive emotions towards students with ADHD can be explained by the hypothesis that some of our participants looked at the students with ADHD not through the perspective of the disorder, but through the perspective of “exceptionality”. This point of view is in line with Krtkova et al. (2022), Sherman et al. (2006), and Sherman et al. (2006) considered the manifestations of ADHD to be the characteristics of geniuses such as Mozart or Einstein and emphasized the need for a new, more positive view of ADHD.

Altogether, our findings suggest that teachers are not only experiencing either positive or negative emotions towards their ADHD students, but that they often simultaneously experience a mixture of these emotions. These findings are in accordance with Anderson et al. (2017), who further pointed out that teachers also reported ambivalent beliefs and behaviours towards ADHD students and connected it with the teachers´ insufficient knowledge about ADHD. Another possible explanation of this ambivalence might also be contextual factors that may increase or decrease ADHD symptoms. On the one hand, teachers might feel positive emotions towards ADHD students when there are mostly positive contextual factors at work, such as adult supportive attitudes towards ADHD students, strong intellectual functioning or positive self-perceptions of competence of students with ADHD (Dvorsky & Langberg, 2016; Mitchell et al., 2021). On the other hand, negative contextual factors, such as poor parenting (Haack et al., 2016) or intellectual disabilities of students with ADHD (Hastings et al., 2005) may strengthen the teachers´ negative emotions towards these students. In general, our findings of ambivalent and often mixed emotions of teachers support the theory of conflict and closeness (Mason et al., 2017; Pianta & Stuhlman, 2004). In line with this theory, we observed that both strong negative emotions, such as anxiety and anger are associated with conflict, and strong positive emotions, such as joy and satisfaction are associated with the closeness in the teacher-student relationship. Thus, a problematic behaviour of ADHD students might increase the conflict and decrease the closeness in the relationship (and vice versa).

The findings of the present study also point out that despite the presence of ambivalent emotions in the relationship of the teacher and ADHD student, the core of this relationship often consists of a strong social bond which may reduce or increase the quality of the relationship. If negative emotions take control over a teacher’s behaviour, a strong social bond can lead to a vicious circle of mutual accusation, hatred, aggression, and bullying, because the teacher may begin to perceive a student with ADHD as bad and intentionally harmful. This may also later lead to school failure, peer rejection, exclusion, loneliness and low self-esteem of students with ADHD (Ewe, 2019). In contrast, if the teacher enables a free passing of their positive emotions and is able to regulate negative emotions, a strong bond can lead to a deeper empathy and understanding and can help the student to manage the symptoms of ADHD and school demands. This is in line with Bergin and Bergin (2009) who suggested the associations between a secure and insecure attachment of teachers and school success and the social competence of students with ADHD.

### Teachers’ beliefs about ADHD and their consequences for the quality of the relationship with ADHD students

Our findings also suggest that the teachers’ attitudes towards ADHD students are often burdened by the teachers’ beliefs that some of those students justified their selfish and oppositional behaviour and the inability to learn by suffering from ADHD. In such cases, some participants were hesitant about whether the ADHD students’ behaviour was due to ADHD or poor parenting. At the same time, the teachers who understood ADHD better were also more tolerant towards their ADHD students. Thus, the participants considered this distinction to be important because it determines the degree of closeness and conflict in the teacher-ADHD student relationship.. Our finding of a conflict between two opposing views of the causes of behaviour in ADHD students is consistent with recent labelling theory (Dauman et al., 2019; Iudici et al., 2014). Dauman et al. (2019) and Iudici et al. (2014) suggested that ADHD label can remove the excessive guilt from students with ADHD, their parents and teachers, which may pave the way for building better relationships. However, excessive perception of causes of disruptive students’ behaviour in neurodevelopmental determinants of ADHD can lead to giving up the effort to manage that behaviour. Thus, too low demands may be required on ADHD students, which does not support their personal growth.

### Participants´ suggestions how to improve the quality of the teacher-ADHD student relationship

Our participants also provided suggestions how to improve the quality of the teacher-ADHD student relationship. This improvement was associated with teacher-student cooperation, viewing the teacher as a positive role model, joint efforts by the teacher and student to find a more adaptive behaviour to ADHD symptoms, open communication about the student’s needs, long-term mutual experience, and using special abilities of students with ADHD for a better learning process. These findings are in accordance with Curtis et al. (2006) who pointed out several categories of special educational needs of students with ADHD, namely a need to receive frequent feedback on undesirable behaviour, to set goals for desirable behaviour, to record the rate of achievement, to reward for strengths and positive behaviours and to be provided with a clear system of evaluation and conduct in the classroom. Merrick (2020) emphasized that open communication in the form of listening to students with ADHD is a suitable way how to get feedback and valuable information about the students’ specific educational needs.

### Strengths and limitations

The main strength of this study is that it is one of a few studies offering insight into the factors that influence the quality of the relationship between the teacher and the student with ADHD. Second, this study is based on the well-established certified DIPEx methodology (Ziebland & McPherson, 2006).

Considering the weaknesses, the social desirability effect was present in the participants’ testimonies, for example, when some participants tried to portray themselves in a good light. The reluctance of self-disclosure in these participants may have impoverished our final findings about the teacher-student with ADHD relationship. It is also possible that these participants tended to respond in a socially desirable way because they were recruited through snowball methods by members of the advisory panel who had the participants in professional care at the time. Thus, though the participants were ensured about the anonymity of the research and the data protection, on a less reflected level they may still have been afraid that the interviewer would reveal something that could ruin their relationship with the members of the advisory panel. Another bias on the side of the advisory board could be that the members have already been working with some participants, so it was easier for them to recommend them for research. Thus, another weakness of the snowball method could be that the participants who were recruited using this method came from a narrow subgroup. However, this weakness was minimized by the fact that the vast majority of the participants were recruited through advertisements in periodicals available in all regions of the Czech Republic.

### Practical implications

The findings of this study suggest several activities that can be used to improve the quality of the teacher-student with ADHD relationship (and thus well-being of the teachers and students with ADHD) and to eliminate the teachers’ stress experienced in teaching ADHD students. These activities may involve the teachers´ education regarding ADHD and their own personal support.

The educational activities may involve refining the teachers´ beliefs and emotional attitudes regarding ADHD students through seminars introducing and explaining the various determinants of ADHD and the relevant methods of working with ADHD students. Teachers may also reduce the level of stress and increase the quality of the relationship with the ADHD students by being prepared for the disruptive behaviour of ADHD students. They should be taught to respond to such behaviour quickly but calmly and to monitor their emotions during the lessons. During emotionally-aroused situations, teachers should try to focus their attention on a possible solution of the problem rather than on their own feelings or on further stirring up negative emotions. Pre-prepared alternatives for solving critical situations in the classroom can also be helpful. Furthermore, teachers should reframe the expectations related to the ADHD students’ school achievement. It cannot be in general expected that the quality and way of school work of students with ADHD can reach the quality of students without ADHD. This means that sometimes even a small school achievement of students with ADHD can be proof of a very efficient work of teachers. If teachers are aware of this, it can protect them from stress, disillusion and disappointments that can arise from holding unrealistic expectations.

Teachers could acquire these skills by attending seminars that could take place one weekend a year at the regional level at selected schools. These seminars could be led by psychologists, teachers, psychotherapists, special pedagogues with many years of experience working with ADHD students and researchers who study ADHD. Those professionals should provide the teachers with theoretical knowledge of ADHD, but also skills on how to build a stable teacher-ADHD student relationship and what methods, approaches and interventions to use when working with those students. The seminar should include exercises in addressing the disruptive behaviour of ADHD students. Professionals in the field of ADHD could imitate the behaviour of ADHD students at school, and teachers should try to respond to this behaviour with skills acquired from the theoretical part of the seminar. At the end of each exercise, there should be a discussion on what the teachers did well and where their skills need to be improved. We are of the opinion that the costs of the seminars would pay off in various forms for the state and schools. The seminars would increase the well-being of teachers, ADHD students, classmates and thus could reduce the state’s financial costs for professional health, psychological and social care.

Furthermore, teachers should be educated in line with European Union and national policies for the education of students with special educational needs (Ministry of Education 2020). These policies recommend the use of adapted textbooks, specific teaching materials, compensatory and rehabilitation equipment and tools, support and behaviour services, additional support staff and additional teaching in specific subjects.

The second set of activities may be represented by the teachers’ psychotherapy, behaviour or supervision, where the teachers may foster their own secure attachment. A secure attachment of the teachers may increase the sense of security and safety of the teachers, which increases resilience in stressful situations in the classroom and thus opens up the teachers’ capacity for a closer and warmer relationship with students with ADHD. Through supervision, the teachers may also refine their motivation for working with ADHD students. This professional support may help teachers to serve as positive role models for ADHD students and to focus on positive skills, traits and talents of students with ADHD.

Future research could focus on a more detailed exploration of the factors that determine the teachers’ ambivalent emotions and the factors that help them to regulate these emotions. Specifically, it can focus on the relationship between the teachers’ ambivalent emotions and contextual factors such as the self-esteem and sense of competence of students with ADHD and teachers.

## Conclusion

The findings of this study suggest that the relationship between a teacher and an ADHD student is characterized by a strong bond despite the presence of ambivalent emotions. Negative emotions were found to be usually caused by the students’ disruptive behaviours driven by ADHD symptoms, by the long-term overload and stress of the teachers and their belief that the disruptive behaviour of ADHD students is intentional, and by abusing the ADHD diagnosis by ADHD students.

Positive emotions were mainly related to the teachers’ efforts to understand the symptoms of ADHD and their knowledge in this area and sometimes elicited by the liveliness and creativity of the students with ADHD. Furthermore, an important role was also played by the teachers’ regulation of the ambivalent emotions, their ability to perceive positive qualities, talents and potentials of students with ADHD, motivation to manage teaching students with ADHD, and interventions that respect special educational needs of students with ADHD.

Based on our findings, we recommend that teachers should complete seminars and trainings focused on theoretical knowledge of ADHD and practical skills on how to work and build stable and warm relationships with ADHD students. Furthermore, teachers could be recommended counselling, supervision or psychotherapy, where they can strengthen their secure attachment.

## Supplementary Material

Supplemental Material

## Additional note

The article is the authors’ original work, has not received prior publication and is not under consideration for publication elsewhere.

## Permission note

All material in the manuscript is original content.
