# Supplementary material for: Ambivalent bonds, positive and negative emotions, and expectations in teachers’ perceptions of relationship with their students with ADHD
Source: Int J Qual Stud Health Well-being. 2022 Jun 16;17(1):2088456. doi: 10.1080/17482631.2022.2088456 (PMC9225763; doi:10.1080/17482631.2022.2088456)
Supplement: Supplemental Material [file ZQHW_A_2088456_SM9720.docx]

**Supplement 1.** Scenario items and sub-items for interview with teachers working with students with ADHD

| **The main items of the scenario** | **Scenario sub-items** | **Research studies that inspired the scenario items** |
| --- | --- | --- |
| Experience of teaching students with ADHD | Please tell me about your experience teaching students with ADHD | (Anderson et al., 2017; Jewers, 2012; Lee & Witruk, 2016; Merrick, 2020; Poznanski et al., 2018). |
| Pedagogical approach | What is it like to be a teacher of a student with ADHD?  How is the approach to students with ADHD different from other students?  How did it influence your teaching?  Is there any positive potential of ADHD? What is the most difficult thing for you in educating these students?  What helps you in teaching students with ADHD? | (Brock et al., 2009; Kauffman & Hornby, 2020; Masse et al., 2015; Merrick, 2020; Serrano, 2015; Smith & Bell, 2015; Thomopoulou, 2013; Trampush et al., 2009) |
| Behaviour of students with ADHD | How does ADHD influence the behaviour of your ADHD students?  How do you respond to ADHD-related behaviours and symptoms?  Do you feel that ADHD somehow limits the students, that it complicates their life? If so, how? On the contrary, does it bring some positives to their life? | (Dourish et al., 2016; Greene et al., 2002; Jansen et al., 2017; Masse et al., 2015; Schulz-Zhecheva et al., 2019; Shoham et al.; Spencer et al., 1998; Suades-Gonzalez et al., 2017; Velki & Dudas, 2016; Velki et al., 2019; Whalen & Henker, 1985) |
| The relationship between teacher and student with ADHD | How would you characterize your students with ADHD? How would you characterize your relationship? Do you understand each other? What is the most difficult thing for you in the relationship? What do you value about this relationship?  Do you think that ADHD diagnosis influences your relationship with students?  Are you talking to your student about ADHD? If so, how?  What does your mutual communication look like? | (Anderson et al., 2017; Becker et al., 2013; Bergin & Bergin, 2009; Feder et al., 2017; Greene et al., 2002; Gumustas & Yulaf, 2019; Honkasilta et al., 2016; Metzger & Hamilton, 2021; Rampp et al., 2020; Sempio et al., 2016) |
| Professional readiness | Did your formal (university) study prepare you for working with a child with ADHD?  What information did you receive about the specific educational needs of students with ADHD?  Were there enough? Were they understandable?  Who did you get this information from?  Where do you get information about working with students with ADHD?  Did you look for any more information? Were you advised where to look for information?  Do you have any questions about ADHD that have not been answered satisfactorily?  Is there any information about ADHD that you would like to know from the experience of other teachers? | (Barr, 2008; Blatchford et al., 2012; Dudova & Kocourkova, 2013; Ewe, 2019; Greene et al., 1997; Greenway & Edwards; Jewers, 2012; Mohr-Jensen et al., 2019; Murtani et al., 2020; Slavicek, 2009; Toye et al., 2019; Wienen et al., 2019; Zendarski et al., 2020) |
| Coping with work with students with ADHD | How do you manage teaching and communication with a student with ADHD?  What helps you manage the teaching of students with ADHD? What makes it difficult for you?  How did you experience finding out that you would have students with ADHD in the classroom? | (Capodieci et al., 2019; Curtis et al., 2006; Dahl et al., 2020; Mao & Shen, 2019; Mohr-Jensen et al., 2019; Moore et al., 2017; Slavicek, 2009; Wienen et al., 2019) |
| Relationships in the classroom, class team  Teaching assistant  School and colleagues | How would you generally describe the functioning of students with ADHD in a class group?  How do classmates approach students with ADHD?  Does this student have friends? What is their relationship?  Do classmates know that some students have ADHD? Are you talking to them about it?  What are the relationships of your students with ADHD with other classmates? Does the diagnosis of ADHD somehow influence relationships with classmates?  Have you encountered bullying during your internship? What role did students with ADHD play in it?  How do you perceive the usefulness of teaching assistants?  What are your colleagues' attitudes towards students with ADHD?  Do you communicate with colleagues about students with ADHD? | (Becker et al., 2013; Bergin & Bergin, 2009; Ewe, 2019; Gardner & Gerdes, 2015; Johnston et al., 2018; Metzger & Hamilton, 2021; O'Driscoll et al., 2012)  (Greenway & Edwards; Miller et al., 2004)  (Karhu et al., 2018; Malmqvist & Nilholm, 2016; Margalit et al., 2020; Toye et al., 2019) |
| Communication with parents | What is your relationship with the parents of students with ADHD?  What is your experience with communication with the parents of students with ADHD? | (Slavicek, 2009; Wolraich et al., 2004) |
| Messages to others | Is there anything else you would like to say? To return to something, to supplement, or to put it in perspective?  What would you say to other teachers who have students with ADHD?  What would you say to the parents of these students?  What would you say to the psychologists and psychiatrists who work with these students?  What would you say to people who have someone around you who have students with ADHD?  What would you say to students with ADHD? | (Bell et al., 2011; Craig et al., 2020; Dahl et al., 2020; Dauman et al., 2019; Finzi-Dottan et al., 2011; Gumustas & Yulaf, 2019; Kellison et al., 2010; Mao & Shen, 2019; Metzger & Hamilton, 2021; Moen et al., 2011; Morris et al., 2019; Varma & Wiener, 2020) |
|  |  |  |

References:

Anderson, D. L., Watt, S. E., & Shanley, D. C. (2017). Ambivalent attitudes about teaching children with attention deficit/hyperactivity disorder (ADHD). *Emotional and Behavioural Difficulties*, *22*(4), 332-349. <https://doi.org/10.1080/13632752.2017.1298242>

Barr, H. (2008). Understanding and Supporting Children with ADHD: Strategies for teachers, parents and other professionals. *Journal of Interprofessional Care*, *22*(6), 673-674. <https://doi.org/10.1080/13561820802379458>

Becker, S. P., Fite, P. J., Luebbe, A. M., Stoppelbein, L., & Greening, L. (2013). Friendship Intimacy Exchange Buffers the Relation between ADHD Symptoms and Later Social Problems among Children Attending an After-School Care Program. *Journal of Psychopathology and Behavioral Assessment*, *35*(2), 142-152. <https://doi.org/10.1007/s10862-012-9334-1>

Bell, L., Long, S., Garvan, C., & Bussing, R. (2011). The impact of teacher credentials on ADHD stigma perceprions. *Psychology in the Schools*, *48*(2), 184-197. <https://doi.org/10.1002/pits.20536>

Bergin, C., & Bergin, D. (2009). Attachment in the Classroom. *Educational Psychology Review*, *21*(2), 141-170. <https://doi.org/10.1007/s10648-009-9104-0>

Blatchford, P., Russell, A., & Webster, R. (2011). *Reassessing the Impact of Teaching Assistants: How research challenges practice and policy (1st ed.)*. Routledge. https://doi.org/10.4324/9780203151969

Brock, S. E., Jimerson, S. R., & Hansen, R. L. (2009). *Identifying, Assessing, and Treating ADHD at School.* Springer US. <https://doi.org/10.1007/978-1-4419-0501-7>

Capodieci, A., Rivetti, T., & Cornoldi, C. (2019). A Cooperative Learning Classroom Intervention for Increasing Peer's Acceptance of Children With ADHD. *Journal of Attention Disorders*, *23*(3), 282-292. <https://doi.org/10.1177/1087054716666952>

Craig, F., Savino, R., Fanizza, I., Lucarelli, E., Russo, L., & Trabacca, A. (2020). A systematic review of coping strategies in parents of children with attention deficit hyperactivity disorder (ADHD). *Research in Developmental Disabilities*, *98*, 103571. <https://doi.org/10.1016/j.ridd.2020.103571>

Curtis, D. F., Pisecco, S., Hamilton, R. J., & Moore, D. W. (2006). Teacher perceptions of classroom interventions for children with ADHD: A cross-cultural comparison of teachers in the United States and New Zealand. *School Psychology Quarterly*, *21*(2), 171-196. <https://doi.org/10.1521/scpq.2006.21.2.171>

Dahl, V., Ramakrishnan, A., Spears, A. P., Jorge, A., Lu, J. N., Bigio, N. A., & Chacko, A. (2020). Psychoeducation Interventions for Parents and Teachers of Children and Adolescents with ADHD: a Systematic Review of the Literature. *Journal of Developmental and Physical Disabilities*, *32*(2), 257-292. <https://doi.org/10.1007/s10882-019-09691-3>

Dauman, N., Haza, M., & Erlandsson, S. (2019). Liberating parents from guilt: a grounded theory study of parents' internet communities for the recognition of ADHD. *International Journal of Qualitative Studies on Health and Well-Being*, *14*(1), 1564520. <https://doi.org/10.1080/17482631.2018.1564520>

Dourish, C., Kaisari, P., & Higgs, S. (2016). Discovery of the First Evidence for a Direct Association Between the Inattentive Symptoms of Attention Deficit Hyperactivity Disorder (ADHD) and Binge Eating: Mediation by Mood and Eating in Response to Internal Hunger and Satiety Signals. *Neuropsychopharmacology*, *41*, 351-352.

Dudova, I., & Kocourkova, J. (2013). ADHD as a source of controversy - unambiguous attitudes or cooperation? *Ceskoslovenska Psychologie*, *57*(2), 190-197.

Ewe, L. P. (2019). ADHD symptoms and the teacher-student relationship: a systematic literature review. *Emotional and Behavioural Difficulties*, *24*(2), 136-155. <https://doi.org/10.1080/13632752.2019.1597562>

Feder, K. M., Bak, C. K., Petersen, K. S., Vardinghus-Nielsen, H., & Kristiansen, T. M. (2017). An ethnographic field study of the influence of social interactions during the school day for children diagnosed with ADHD. *European Journal of Special Needs Education*, *32*(3), 406-421. <https://doi.org/10.1080/08856257.2016.1260207>

Finzi-Dottan, R., Triwitz, Y. S., & Golubchik, P. (2011). Predictors of stress-related growth in parents of children with ADHD. *Research in Developmental Disabilities*, *32*(2), 510-519. <https://doi.org/10.1016/j.ridd.2010.12.032>

Gardner, D. M., & Gerdes, A. C. (2015). A Review of Peer Relationships and Friendships in Youth With ADHD. *Journal of Attention Disorders*, *19*(10), 844-855. <https://doi.org/10.1177/1087054713501552>

Greene, R. W., Abidin, R. R., & Kmetz, C. (1997). The Index of Teaching Stress: A measure of student-teacher compatibility. *Journal of School Psychology*, *35*(3), 239-259. <https://doi.org/10.1016/s0022-4405(97)00006-x>

Greene, R. W., Beszterczey, S. K., Katzenstein, T., Park, K., & Goring, J. (2002). Are students with ADHD more stressful to teach? Patterns of teacher stress in an elementary school sample. *Journal of Emotional and Behavioral Disorders*, *10*(2), 79-89. <https://doi.org/10.1177/10634266020100020201>

Greenway, C. W., & Edwards, A. R. Knowledge and attitudes towards attention-deficit hyperactivity disorder (ADHD): a comparison of teachers and teaching assistants. *Australian Journal of Learning Difficulties*. <https://doi.org/10.1080/19404158.2019.1709875>

Gumustas, F., & Yulaf, Y. (2019). Effects of parents' attachment styles and attention deficit symptoms on social responsiveness in children with ADHD. *Anadolu Psikiyatri Dergisi-Anatolian Journal of Psychiatry*, *20*(6), 651-658. <https://doi.org/10.5455/apd.30826>

Honkasilta, J., Vehkakoski, T., & Vehmas, S. (2016). 'The teacher almost made me cry' Narrative analysis of teachers' reactive classroom management strategies as reported by students diagnosed with ADHD. *Teaching and Teacher Education*, *55*, 100-109. <https://doi.org/10.1016/j.tate.2015.12.009>

Jansen, D., Petry, K., Ceulemans, E., van der Oord, S., Noens, I., & Baeyens, D. (2017). Functioning and participation problems of students with ADHD in higher education: which reasonable accommodations are effective? *European Journal of Special Needs Education*, *32*(1), 35-53. <https://doi.org/10.1080/08856257.2016.1254965>

Jewers, R. (2012). Teaching Teens with ADD, ADHD & Executive Function Deficits: A Quick Reference Guide for Teachers and Parents, 2nd Edition. *Canadian Journal of Occupational Therapy-Revue Canadienne D Ergotherapie*, *79*(3), 158-158. <https://doi.org/10.1177/000841741207900301>

Johnston, O. G., Derella, O. J., & Burke, J. D. (2018). Identification of Oppositional Defiant Disorder in Young Adult College Students. *Journal of Psychopathology and Behavioral Assessment*, *40*(4), 563-572. <https://doi.org/10.1007/s10862-018-9696-0>

Karhu, A., Narhi, V., & Savolainen, H. (2018). Inclusion of pupils with ADHD symptoms in mainstream classes with PBS. *International Journal of Inclusive Education*, *22*(5), 475-489. <https://doi.org/10.1080/13603116.2017.1370741>

Kauffman, J. M., & Hornby, G. (2020). Inclusive Vision Versus Special Education Reality. *Education Sciences*, *10*(9), 258. <https://doi.org/10.3390/educsci10090258>

Kellison, I., Bussing, R., Bell, L., & Garvan, C. (2010). Assessment of stigma associated with attention-deficit hyperactivity disorder: Psychometric evaluation of the ADHD Stigma Questionnaire. *Psychiatry Research*, *178*(2), 363-369. <https://doi.org/10.1016/j.psychres.2009.04.022>

Lee, Y., & Witruk, E. (2016). Teachers' knowledge, perceived teaching efficacy, and attitudes regarding students with ADHD: a cross-cultural comparison of teachers in South Korea and Germany. *Health Psychology Report*, *4*(2), 103-115. <https://doi.org/10.5114/hpr.2016.58383>

Malmqvist, J., & Nilholm, C. (2016). The antithesis of inclusion? The emergence and functioning of ADHD special education classes in the Swedish school system. *Emotional and Behavioural Difficulties*, *21*(3), 287-300. <https://doi.org/10.1080/13632752.2016.1165978>

Mao, S. J., & Shen, J. (2019). What should professionals do for the parents of children with ADHD? *World Journal of Pediatrics*, *15*(6), 620-621. <https://doi.org/10.1007/s12519-019-00231-9>

Margalit, M., Abramowitz, M. Z., Jaffe, E., Herbst, R., & Knobler, H. Y. (2020). Inclusion in community services and PTSD symptoms among adolescents with attention-deficit disorders (ADHD) and learning disabilities (LD). *European Journal of Special Needs Education*, *35*(4), 482-496. <https://doi.org/10.1080/08856257.2019.1708640>

Masse, L., Begin, J. Y., Couture, C., Plouffe-Leboeuf, T., Beaulieu-Lessard, M., & Tremblay, J. (2015). Teachers' stress about the integration of students with behavioral problems. *Education Et Francophonie*, 43(2), 179-200. <https://doi.org/10.7202/1034491ar>

Merrick, R. (2020). Pupil participation in planning provision for special educational needs: teacher perspectives. *Support for Learning*, *35*(1), 101-118. <https://doi.org/10.1111/1467-9604.12288>

Metzger, A. N., & Hamilton, L. T. (2021). The Stigma of ADHD: Teacher Ratings of Labeled Students. *Sociological Perspectives*, *64*(2), 258-279. <https://doi.org/10.1177/0731121420937739>

Miller, M. L., Fee, V. E., & Jones, C. J. (2004). Psychometric properties of ADHD rating scales among children with mental retardation II: Validity. *Research in Developmental Disabilities*, *25*(5), 477-492. <https://doi.org/10.1016/j.ridd.2003.11.002>

Moen, O. L., Hall-Lord, M. L., & Hedelin, B. (2011). Contending and Adapting Every Day: Norwegian Parents' Lived Experience of Having a Child With ADHD. *Journal of Family Nursing*, *17*(4), 441-462. <https://doi.org/10.1177/1074840711423924>

Mohr-Jensen, C., Steen-Jensen, T., Bang-Schnack, M., & Thingvad, H. (2019). What Do Primary and Secondary School Teachers Know About ADHD in Children? Findings From a Systematic Review and a Representative, Nationwide Sample of Danish Teachers. *Journal of Attention Disorders*, *23*(3), 206-219. <https://doi.org/10.1177/1087054715599206>

Moore, D. A., Russell, A. E., Arnell, S., & Ford, T. J. (2017). Educators' experiences of managing students with ADHD: a qualitative study. *Child Care Health and Development*, *43*(4), 489-498. <https://doi.org/10.1111/cch.12448>

Morris, S. H., Nahmias, A., Nissley-Tsiopinis, J., Orapallo, A., Power, T. J., & Mautone, J. A. (2019). Research to Practice: Implementation of Family School Success for Parents of Children With ADHD. *Cognitive and Behavioral Practice*, *26*(3), 535-546. <https://doi.org/10.1016/j.cbpra.2019.03.002>

Murtani, B. J., Wibowo, J. A., Liu, C. A., Goey, M. R., Harsono, K., Mardani, A. A. P., & Wiguna, T. (2020). Knowledge/understanding, perception and attitude towards attention-deficit/hyperactivity disorder (ADHD) among community members and healthcare professionals in Indonesia. *Asian Journal of Psychiatry*, *48*, 101912. <https://doi.org/10.1016/j.ajp.2019.101912>

O'Driscoll, C., Heary, C., Hennessy, E., & McKeague, L. (2012). Explicit and implicit stigma towards peers with mental health problems in childhood and adolescence. *Journal of Child Psychology and Psychiatry*, *53*(10), 1054-1062. <https://doi.org/10.1111/j.1469-7610.2012.02580.x>

Poznanski, B., Hart, K. C., & Cramer, E. (2018). Are Teachers Ready? Preservice Teacher Knowledge of Classroom Management and ADHD. *School Mental Health*, *10*(3), 301-313. <https://doi.org/10.1007/s12310-018-9259-2>

Rampp, G., Roesler, C., & Peter, J. (2020). Attachment Representations, Critical Life Events and ADHD in Boys at 6 to 10 Years of Age. *Praxis Der Kinderpsychologie Und Kinderpsychiatrie*, *69*(1), 40-59. <https://doi.org/10.13109/prkk.2020.69.1.40>

Schulz-Zhecheva, Y., Voelkle, M., Beauducel, A., Buch, N., Fleischhaker, C., Bender, S., Saville, C. W. N., Biscaldi, M., & Klein, C. (2019). ADHD Traits in German School-Aged Children: Validation of the German Strengths and Weaknesses of ADHS Symptoms and Normal Behavior (SWAN-DE) Scale. *Journal of Attention Disorders*, *23*(6), 553-562. <https://doi.org/10.1177/1087054716676365>

Sempio, O. L., Fabio, R. A., Tiezzi, P., & Cedro, C. (2016). Parental and teachers attachment in children at risk of ADHD and with ADHD. *Life Span and Disability*, *19*(1), 57-77.

Serrano, M. A. (2015). Successful educational actions for inclusion and social cohesion in Europe. *Revista Internacional De Organizaciones*(14), 161-164.

Shoham, R., Sonuga-Barke, E., Yaniv, I., & Pollak, Y. ADHD Is Associated With a Widespread Pattern of Risky Behavior Across Activity Domains. *Journal of Attention Disorders*, 1087054719875786. <https://doi.org/10.1177/1087054719875786>

Slavicek, M. (2009). Understanding of children with ADHD syndrome and support strategies for teachers, parents and other professionals. *Suvremena Psihologija*, *12*(2), 439-442.

Smith, A., & Bell, S. (2015). Towards Inclusive Learning Environments (TILE): Developing the 'Roadmap for the Inclusion of Students with Special Educational Needs in Vocational Education and Workplace Settings'. *Support for Learning*, *30*(2), 150-160. <https://doi.org/10.1111/1467-9604.12082>

Spencer, T., Biederman, J., Wilens, T. E., & Faraone, S. V. (1998). Adults with attention-deficit/hyperactivity disorder: A controversial diagnosis. *Journal of Clinical Psychiatry*, *59 (7)*, 59-68.

Suades-Gonzalez, E., Forns, J., Garcia-Esteban, R., Lopez-Vicente, M., Esnaola, M., Alvarez-Pedrerol, M., Sunyer, J. (2017). A Longitudinal Study on Attention Development in Primary School Children with and without Teacher-Reported Symptoms of ADHD. *Frontiers in Psychology*, *8*, 655. <https://doi.org/10.3389/fpsyg.2017.00655>

Thomopoulou, V. (2013, Jul 01-03). Special education in Greece and Europe - the necessity of inclusion *EDULEARN Proceedings* [Edulearn13: 5th international conference on education and new learning technologies]. 5th International Conference on Education and New Learning Technologies (EDULEARN), Barcelona, SPAIN.

Toye, M. K., Wilson, C., & Wardle, G. A. (2019). Education professionals' attitudes towards the inclusion of children with ADHD: the role of knowledge and stigma. *Journal of Research in Special Educational Needs*, *19*(3), 184-196. <https://doi.org/10.1111/1471-3802.12441>

Trampush, J. W., Miller, C. J., Newcorn, J. H., & Halperin, J. M. (2009). The Impact of Childhood ADHD on Dropping Out of High School in Urban Adolescents/Young Adults. *Journal of Attention Disorders*, *13*(2), 127-136. <https://doi.org/10.1177/1087054708323040>

Varma, A., & Wiener, J. (2020). Perceptions of ADHD Symptoms in Adolescents With Attention-Deficit/Hyperactivity Disorder: Attributions and Stigma. *Canadian Journal of School Psychology*, *35*(4), 252-265. <https://doi.org/10.1177/0829573520936459>

Velki, T., & Dudas, M. (2016). Do more hyperactive children show more symptoms of agression? *Ljetopis Socijalnog Rada*, *23*(1), 87-121. <https://doi.org/10.3935/ljsr.v23i1.93>

Velki, T., Uzarevic, Z., & Dubovicki, S. (2019). Self-evaluated ADHD symptoms as risk adaptation factors in elementary school children. *Drustvena Istrazivanja*, *28*(3), 503-522. <https://doi.org/10.5559/di.28.3.07>

Whalen, C. K., & Henker, B. (1985). The social worlds of hyperactive (ADDH) children. *Clinical Psychology Review*, *5*(5), 447-478. <https://doi.org/10.1016/0272-7358(85)90004-2>

Wienen, A. W., Sluiter, M. N., Thoutenhoofd, E., de Jonge, P., & Batstra, L. (2019). The advantages of an ADHD classification from the perspective of teachers. *European Journal of Special Needs Education*, *34*(5), 649-662. <https://doi.org/10.1080/08856257.2019.1580838>

Wolraich, M. L., Bickman, L., Lambert, E. W., Simmons, T., & Doffing, M. (2004). Improving teacher, parent & physician communication in the management of children with ADHD. *Pediatric Research*, *55*(4), 74A-74A.

Zendarski, N., Haebich, K., Bhide, S., Quek, J., Nicholson, J. M., Jacobs, K. E., Efron, D., & Sciberras, E. (2020). Student-teacher relationship quality in children with and without ADHD: A cross-sectional community based study. *Early Childhood Research Quarterly*, *51*, 275-284. <https://doi.org/10.1016/j.ecresq.2019.12.006>
